# Erythrocyte Fragility in Progressive Multiple Sclerosis

**DOI:** 10.1111/ene.70595

**Published:** 2026-04-17

**Authors:** Carmen Jacob, Thomas E. Williams, Charlotte M. Stuart, Aviva Witkover, Simon Hametner, Hans Lassmann, Charles R. M. Bangham, Jeremy Chataway, Ian Galea

**Affiliations:** ^1^ Clinical Neurosciences, Clinical & Experimental Sciences, Faculty of Medicine University of Southampton Southampton UK; ^2^ Wessex Neurological Centre, Southampton General Hospital University Hospital Southampton NHS Foundation Trust Southampton UK; ^3^ Queen Square Multiple Sclerosis Centre, Department of Neuroinflammation, UCL Queen Square Institute of Neurology, Faculty of Brain Sciences University College London London UK; ^4^ Department of Infectious Diseases, Faculty of Medicine Imperial College London London UK; ^5^ Division of Neurochemistry and Neuropathology Medical University of Vienna Vienna Austria; ^6^ Department of Neuroimmunology, Center for Brain Research Medical University of Vienna Vienna Austria; ^7^ National Institute for Health Research, Biomedical Research Centre University College London Hospitals London UK

**Keywords:** erythrocyte, haemoglobin, haemolysis, multiple sclerosis, osmotic fragility

## Abstract

**Background:**

Aberrant iron homeostasis is increasingly recognized as a key pathological feature in progressive multiple sclerosis (MS). Although the source of excess brain iron remains unclear, haemoglobin is one possible source. To test this hypothesis, we conducted a case–control study to determine whether erythrocytes are more fragile in people with progressive MS (PwPMS) and examined associations between erythrocyte fragility and brain atrophy.

**Methods:**

PwPMS and control individuals were recruited from two centres. Two measures of erythrocyte fragility were assessed at baseline: the Median Corpuscular Fragility (MCF) and haemolysis curve slope. A subset of PwPMS in one centre underwent MR imaging at the same time as osmotic fragility testing and annually for three years thereafter.

**Results:**

A total of 174 participants were included (75 PwPMS, 99 controls), with MRI data available for 44 PwPMS. No significant differences in the MCF were observed between PwPMS and controls in either the full or age‐matched cohorts. However, the haemolysis curve slope in PwPMS was less steep than healthy controls (median PwPMS = −24.76, controls = −28.73, *p* = 0.017), consistent with a subpopulation of fragile erythrocytes, with a similar trend in the age‐matched subset (*p* = 0.056). Erythrocyte fragility was associated with normalized whole brain volume at the time of osmotic fragility testing and up to three years thereafter.

**Conclusions:**

Extracellular haemoglobin from lysis of an erythrocyte subpopulation may contribute to neurodegeneration in progressive MS. Further research is warranted to elucidate the interplay between erythrocyte health, inflammation and neurodegeneration, which may open avenues for novel therapeutic strategies.

## Graphical abstract

While the factors contributing to progression in multiple sclerosis (MS) are not fully understood, circulating extracellular haemoglobin was found to be associated with brain atrophy. In this case–control study of 174 people, erythrocytes were more fragile in progressive MS compared with controls, and greater fragility was linked to lower brain volume over three years. These findings suggest a causal chain of events linking erythrocyte fragility, haemolysis, haemoglobin neurotoxicity and brain atrophy.

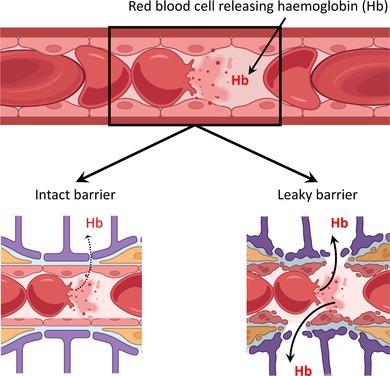

## 1 Introduction

Despite the availability of effective disease‐modifying therapies for relapsing–remitting multiple sclerosis (RRMS), therapeutic progress in progressive forms of the disease remains limited. Although the approval of ocrelizumab for primary progressive MS (PPMS) and siponimod for secondary progressive MS (SPMS) marks a significant advance, both agents are restricted to patients with evidence of ongoing inflammatory activity. Currently, no licenced treatments exist for inactive progressive MS. A major barrier to developing neuroprotective therapies is the incomplete understanding of progressive MS pathophysiology. Emerging evidence implicates aberrant iron homeostasis within the central nervous system (CNS) in neurodegenerative disease [1], either by having direct pathological effects or by acting as a marker for altered iron handling/exposure to iron–metalloproteins.

In people with MS (PwMS), histological and MRI studies reveal altered iron distribution in the brain. Iron concentrations are reduced in some areas such as normal‐appearing white matter (NAWM), thalamus and chronic demyelinated lesions, but elevated in perilesional rims and deep grey matter [2, 3, 4, 5, 6, 7]. High deep grey matter iron concentrations correlate with longer disease duration and greater disability [8, 9]. Perilesional iron is predominantly contained within activated microglia/macrophages exhibiting a proinflammatory phenotype and is associated with lesion expansion [2, 4, 5, 6, 10, 11]. While the reason for these altered iron concentrations remains to be determined, it may include contributions from: (1) iron redistribution secondary to oligodendrocyte and myelin damage; (2) deposition of blood‐derived iron; (3) altered iron concentrations secondary to tissue atrophy.

In prior work, we demonstrated that an increase in circulating free haemoglobin (Hb) over two years was independently associated with accelerated brain atrophy in a cohort of 140 patients with SPMS [12]. Studies have reported increased erythrocyte fragility in MS [13, 14, 15], as well as a number of other changes to erythrocytes, such as increased diameter [16], impaired erythrocyte antioxidant capacity [17, 18, 19], altered rheological features [20, 21] and anisocytosis (morphological heterogeneity) as measured by red cell distribution width [22]. MS lesions are closely associated with cerebral vasculature, and iron accumulation has been observed in the walls of dilated veins within lesions [23, 24], as reviewed by Bamm and Harauz [25]. In patients with early MS, higher free Hb in the cerebrospinal fluid is associated with the number of cortical lesions, supporting a potentially pathogenic role [26].

These findings suggest that CNS iron deposition in progressive MS may be explained by a soluble iron source originating from the circulation. Given that around 60% of total body iron is contained in Hb [27], even small changes in the rate of erythrocyte turnover have the potential to substantially alter iron homeostasis [25]. Hence, intravascular haemolysis, even at low levels, can release significant quantities of free Hb into the circulation.

Vascular density—and thus the surface area of the blood–brain barrier (BBB) per unit of tissue volume—is approximately four‐fold greater in grey matter than in white matter [28]. The relationship between Hb and tissue alterations in multiple sclerosis (MS) appears more pronounced in grey matter than in white matter [26, 29], supporting a BBB‐dependent mechanism. While focal BBB disruption is well recognized in contrast‐enhancing lesions, diffuse BBB hyper‐permeability also affects NAWM in both relapsing–remitting [30] and progressive forms of MS [31, 32]. In the context of intravascular haemolysis, such BBB disruption may facilitate the extravasation of Hb into the surrounding brain parenchyma, contributing to iron deposition.

In vitro studies have shown that Hb is toxic to oligodendrocytes [33] and oligodendrocyte precursor cells [34]. Hemin, a Hb breakdown product, caused demyelination and axonal loss in a myelinating cell culture [35]. Hb is lethal to neurons at concentrations as low as 10 μM [36], via a number of mechanisms, including oxidative stress and ferroptosis [37]. Moreover, neurons surviving exposure to 10 μM Hb show impaired synaptic function [38].

These findings suggest a pathogenic mechanism starting with increased erythrocyte fragility, chronic low‐grade haemolysis, and elevated concentration of free circulating Hb, which, when coupled with increased BBB permeability, may lead to neurotoxicity and iron deposition. This process may be particularly pronounced in regions of active inflammation, such as MS plaques. If the above mechanism were present, one would expect erythrocyte fragility to be greater in PwMS than in controls, and to be associated with more severe disease outcomes, such as brain atrophy. To test these two hypotheses, we conducted a cross‐sectional case–control study to measure the osmotic fragility of erythrocytes from people with progressive MS (PwPMS).

## 2 Methods

### 2.1 Cohort 1

Patient samples were obtained from participants attending screening and baseline appointments for the MS‐STAT2 trial (NCT03387670), prior to the first dose of study medication. All participants had a diagnosis of SPMS with progression over the preceding two years. Key exclusion criteria included PPMS and recent use of disease modifying therapies (DMT) other than those licensed for SPMS. Control samples were obtained from individuals accompanying patients to trial visits or from research department staff with no known neurological condition. All participants gave written informed consent to a biomarker sub study of the MS‐STAT2 trial (17/LO/1509). Blood samples were obtained through a 21G Vacutainer system to minimize iatrogenic haemolysis. Samples were gently inverted following venepuncture, placed immediately on ice and transported to the laboratory for osmotic fragility testing within three hours of sampling.

Brain MRI was acquired at a single site on a 3 T Philips Ingenia CX MR system. 3D sagittal T1‐weighted magnetization‐prepared rapid acquisition gradient echo and 3D sagittal fluid‐attenuated inversion recovery (FLAIR) images both with 1mm^3^ isotropic voxels were acquired. 2D T1 post‐gadolinium images were additionally collected at month 0 only. Participants recruited into the MRI sub‐study after month 0 or who declined cannulation could participate without post‐gadolinium sequences.

Lesions were automatically segmented via nicMSlesions [39], quality checked, and then used to derive T2 lesion volumes (T2LV). These lesion masks were additionally used for lesion filling on the 3DT1 scans [40]. Brain tissue volume, normalized for subject head size, was estimated with Structural Image Evaluation using Normalization of Atrophy Cross‐sectional, SIENAX [41], part of FSL [42].

For osmotic fragility testing, buffers were prepared in advance using distilled water and 10% NaCl‐PO_4_. The following buffer concentrations were used (% NaCl‐PO_4_): 0.8, 0.7, 0.65, 0.63, 0.6, 0.58, 0.55, 0.53, 0.5, 0.48, 0.45, 0.43, 0.4, 0.35, 0.3, 0.2, 0.1, 0. Buffers were kept refrigerated between use and allowed to warm to room temperature prior to each assay. Twenty μL of blood was added to 1 mL of each buffer, inverted and incubated at room temperature for 30 min. Following incubation, samples were centrifuged at 1200 g for 5 min and 200 μL of supernatant transferred to a 96‐well plate for optical analysis. Each sample was analysed in duplicate.

The extent of haemolysis from each buffer concentration was analysed by assessing the absorbance of each supernatant at 560 nm (representing Hb) using a microplate reader (FLUOstar Omega). The absorbance of supernatant from the 0% buffer (pure distilled water) was set as the 100% haemolysis reference.

### 2.2 Cohort 2

Adult participants were recruited and consented (national research ethics approval 12/SC/0176 and 18/LO/2105; institutional research ethics approval ERGO5562 and ERGO46018). People with progressive MS not receiving DMT were included, but not those with concurrent or recent infection or co‐existing haematological disorders. Exclusion criteria for control individuals were: (1) haematological disorders, (2) autoimmune or active inflammatory disorders, (3) chronic medical conditions which were uncontrolled or active at the time of recruitment. All participants were non‐smokers in the year preceding study participation.

Compared with Cohort 1, more care was taken to reduce the chance of in vitro haemolysis of the samples by careful handling, avoidance of ice, transport of samples in a padded container, proximity of clinic and laboratory, availability of staff on stand‐by to conduct osmotic fragility testing promptly, and short needle‐to‐test time. Blood was drawn with a 21G needle and immediately transported on foot to a laboratory within two minutes' walking distance of the clinic. Erythrocyte osmotic fragility testing was performed immediately on receipt of the sample in most cases and always within 15 min after venesection, using the standardized protocol as in Cohort 1 (see above), described in detail elsewhere [43].

### 2.3 Haemolysis Curve

**FIGURE 1:**
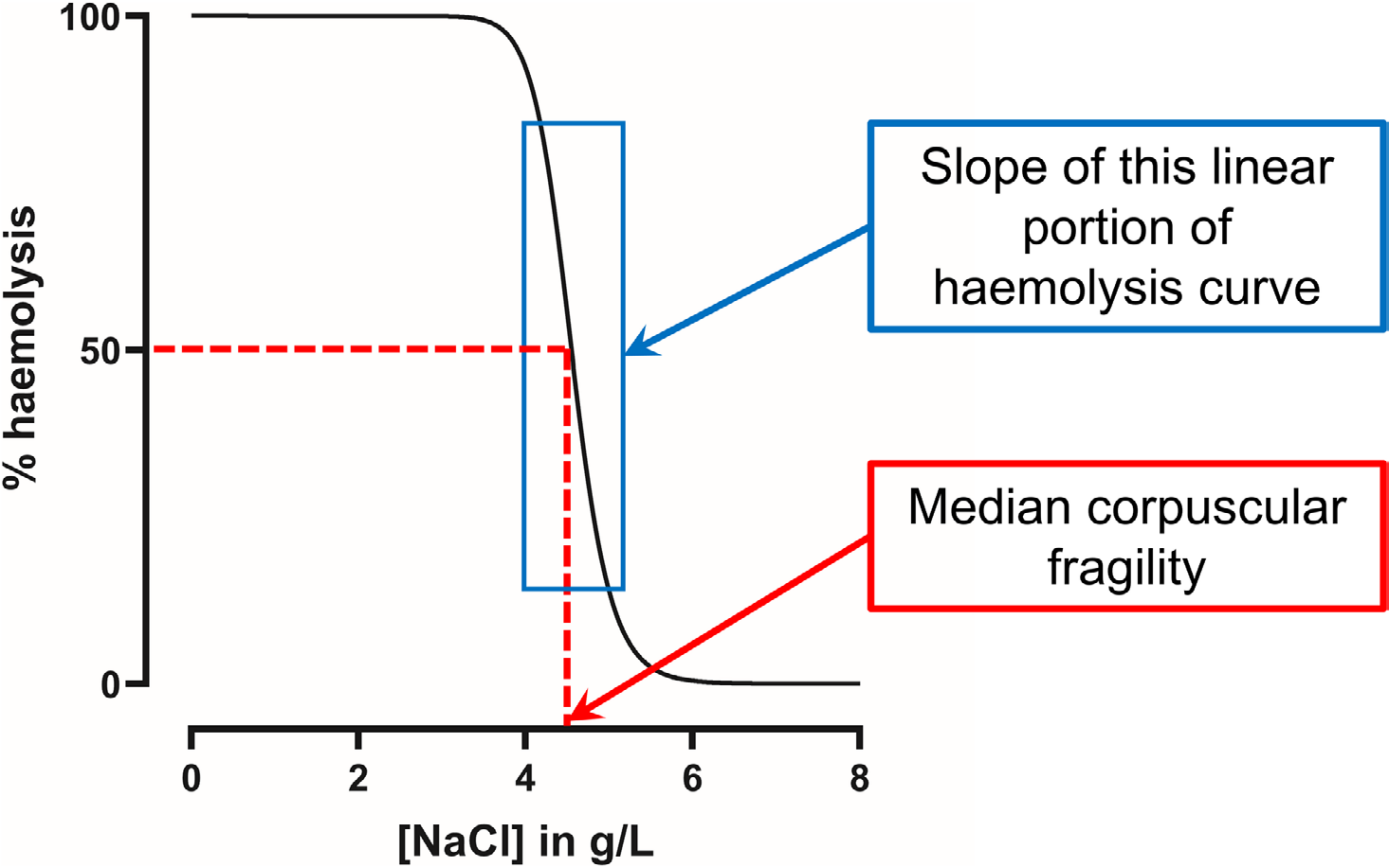
Osmotic fragility curve showing derivation of the two erythrocyte fragility parameters: Median corpuscular fragility and the haemolysis curve slope.

Data from both cohorts were analysed in the same way. A four‐parameter variable slope sigmoidal model was fitted to haemolysis data in GraphPad Prism v10. Haemolysis curves were visually checked by the same operator for both cohorts, and curve fit was adjusted as necessary. Outcome parameters were the IC50, which is the concentration of NaCl‐PO_4_ causing 50% haemolysis (also known as the median corpuscular fragility, MCF), and the slope of the haemolysis curve [44], as visualized in Figure 1. A higher MCF suggests increased fragility, as 50% haemolysis is obtained at a higher NaCl‐PO_4_ concentration, which is a lower osmotic challenge. The slope is always negative and a less steep (i.e., less negative) slope indicates greater dispersion of osmotic fragility across the studied erythrocyte population, and is considered to be a more sensitive measure than the MCF [44].

### 2.4 Outcome Parameters and Statistical Analysis

The primary outcome parameters of erythrocyte fragility were the MCF and the slope of the haemolysis curve, testing the first hypothesis that erythrocytes of people with MS exhibit increased fragility compared with control individuals. The second hypothesis was tested by examining the association between osmotic fragility parameters (MCF and haemolysis curve slope) and whole brain volume measurements in PwPMS from Cohort 1.

Statistical analyses were performed using SPSS v30. Group comparisons employed Pearson's chi‐squared, independent‐samples *t*‐tests or Mann–Whitney *U* tests, depending on data type and distribution. Imaging analyses were conducted using linear regression models, where the aim was to test for a biological association of erythrocyte fragility with whole brain volume, rather than explaining the maximum variance in whole brain volume for prediction purposes. Confounders were selected according to the minimal sufficient adjustment set identified from the directed acyclic graph. Variables that lay on common back‐door paths between erythrocyte fragility and whole brain volume (age and T2 lesion volume) were included in the adjustment set to block non‐causal associations, whereas other covariates were not adjusted for. This resulted in a covariate to sample size ratio of 1:10, which is acceptable to avoid over‐fitting. Results were considered statistically significant if the *p* value was less than 0.05. In figures, *p* values ≥ 0.1 are not shown, *p* values between 0.05 and 0.1 are shown, and *p* values < 0.05 are indicated by a star system. In tables, all *p* values are shown.

## 3 Results

### 3.1 Study Population

Participant characteristics are summarized in Table 1. In both cohorts, control individuals were significantly younger than PwPMS. Sex distribution did not differ significantly between cases and controls, although a trend towards higher female representation was observed in the PwPMS groups. Age, but not sex, can affect erythrocyte osmotic fragility; older age is associated with increased fragility [43, 45]. Hence, erythrocyte fragility parameters were also assessed in age‐matched sub‐cohorts: 34 PwPMS and 34 neurologically normal controls (Cohort 1), and 14 PwPMS and 14 healthy controls (Cohort 2).

**TABLE 1:** Participant characteristics in the two cohorts.

	pwPMS	Controls	Significance (*p*)
Cohort 1			
All cases			
*N*	54	47	
Age in years (median and range)	55 (34 to 66)	50 (22 to 66)	* (0.021)^†^
Sex (% female)	72.2%	57.4%	NS (0.120)^‡^
EDSS (median and range)	6.0 (4.0 to 6.5)	—	
MS duration in years (mean ± SD)	21.1 ± 10.2	—	
Age‐matched sub‐cohort			
*N*	34	34	
Age in years (median and range)	54 (34 to 66)	54 (34 to 66)	NS (0.922)^†^
Sex (% female)	64.7%	47.1%	NS (0.143)^‡^
EDSS (median and range)	6.0 (4.0 to 6.5)	—	
MS duration in years (mean ± SD)	21.9 ± 10.8	—	
Cohort 2			
All cases			
*N*	21	52	
Age in years (median and range)	56 (38–68)	45 (19 to 82)	*** (< 0.001)^†^
Gender (% female)	72.4	69.6	NS (*p* = 0.753)^‡^
EDSS (median and range)	6.25 (2.5 to 8.0)		
MS duration in years (mean ± SD)	21.6 ± 12.2		
Age‐matched sub‐cohort			
*N*	14	14	
Age in year (median and range)	55 (38 to 63)	53 (39 to 63)	NS (0.734)^†^
Gender (% female)	57.1%	71.4%	NS (0.430)^‡^
EDSS (median and range)	6.5 (3.0 to 8.0)		
MS duration in years (mean ± SD)	22.77 ± 13.6		

*Note: p* values were calculated using the Mann–Whitney *U* test^†^ for age or Pearson's chi‐squared test^‡^ for gender.

Abbreviations: EDSS, expanded disability status scale; NS, not significant; PMS, progressive multiple sclerosis; PwPMS, people with progressive multiple sclerosis; SD, standard deviation.

### 3.2 Erythrocyte Fragility Parameters Between Cohorts

**FIGURE 2:**
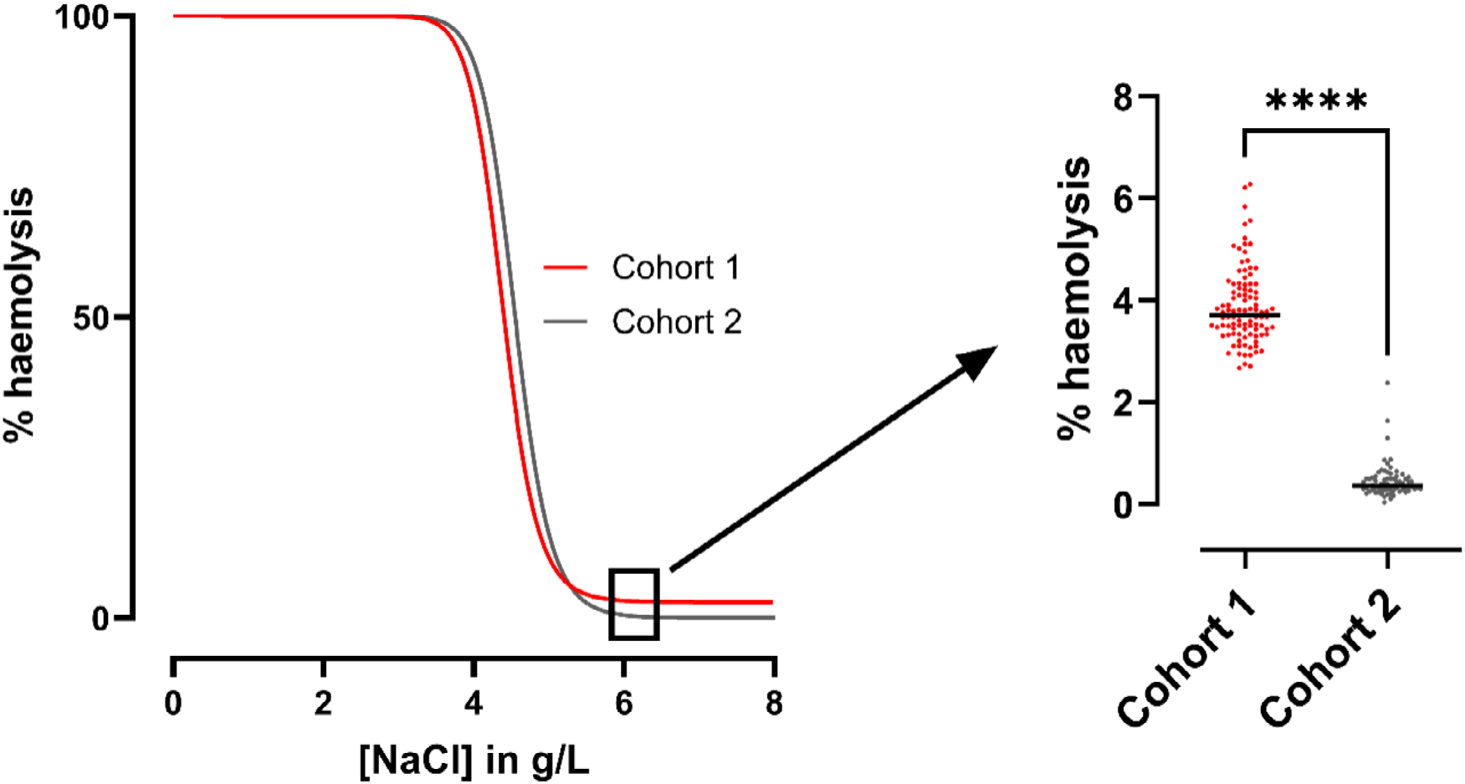
Mean osmotic fragility curves of all participants in Cohorts 1 (red) and 2 (grey). A significant degree of in vitro haemolysis occurred at isotonic or near‐isotonic buffer concentrations in Cohort 1. ****p < 0.0001 (Mann–Whitney *U* test).

**FIGURE 3:**
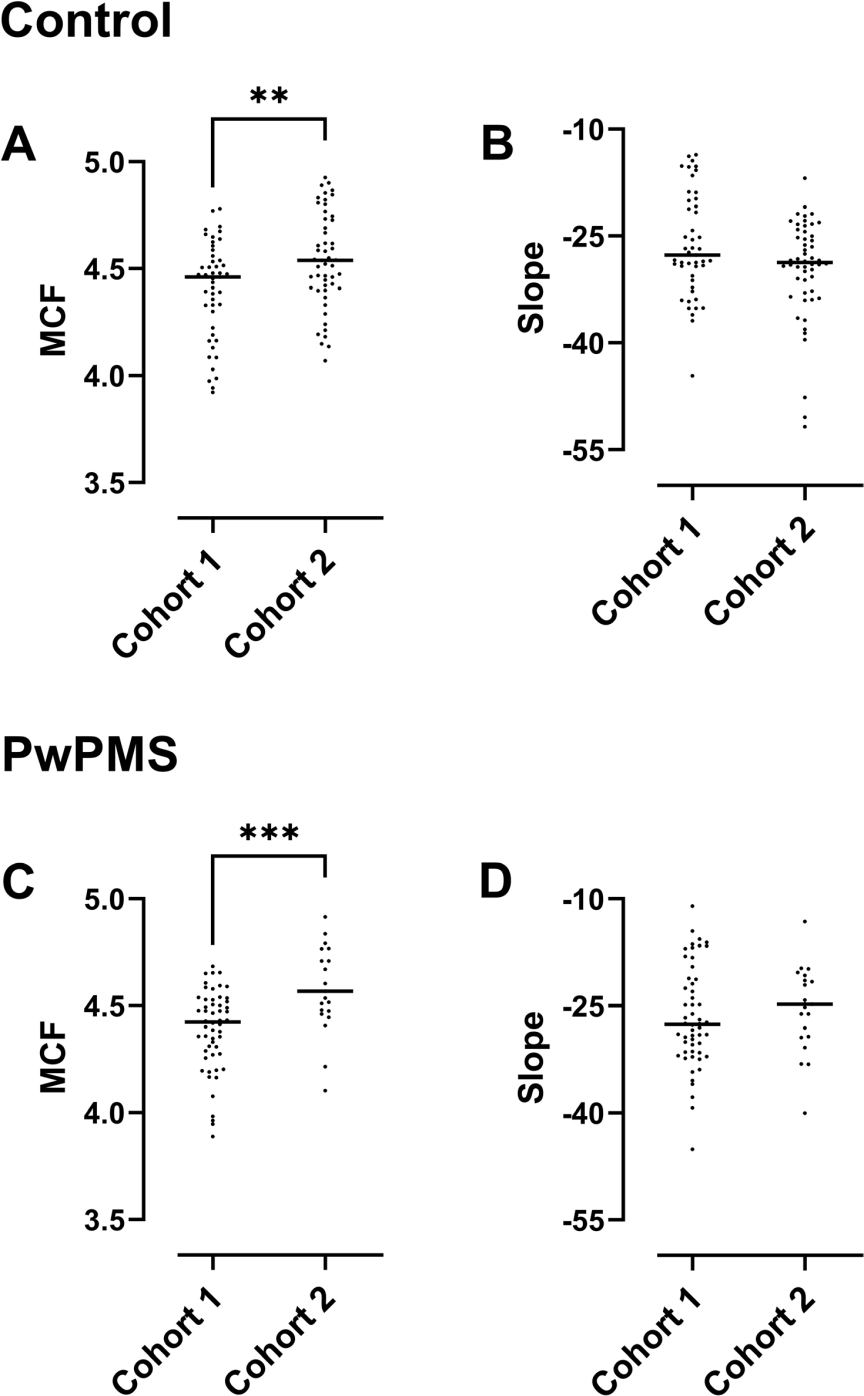
Median corpuscular fragility (A, C) and haemolysis curve slope (B, D) in Cohort 1 versus Cohort 2, within control individuals (A, B) and people with progressive multiple sclerosis (C, D). **p < 0.01, ***p < 0.001, Mann–Whitney *U* tests. MCF, median corpuscular fragility; PwPMS, people with progressive MS.

In Cohort 2 additional precautions were taken to minimize ex vivo haemolysis, to preserve any fragile erythrocyte subpopulations prior to osmotic fragility testing. This resulted in a ten‐fold reduction in the level of in vitro haemolysis in isotonic or near‐isotonic buffer concentrations during testing (*p* < 0.0001, Figure 2). Erythrocyte fragility parameters are compared between cohorts, separately for control and PwPMS subgroups, in Figure 3. Median MCF was higher in Cohort 2 compared with Cohort 1, in both control (4.539 versus 4.460 g/L, *p* = 0.005) and PwPMS groups (4.568 versus 4.423 g/L, *p* < 0.001). The median haemolysis curve slope in PwPMS appeared to be less steep in Cohort 2 versus Cohort 1 (−24.76 versus −27.59), but this was not statistically significant (*p* = 0.4). A higher MCF implies increased fragility, and a less steep haemolysis curve slope indicates greater heterogeneity in erythrocyte fragility (i.e., the presence of a subpopulation of more fragile erythrocytes).

### 3.3 Erythrocyte Fragility Parameters Between Cases and Controls

**FIGURE 4:**
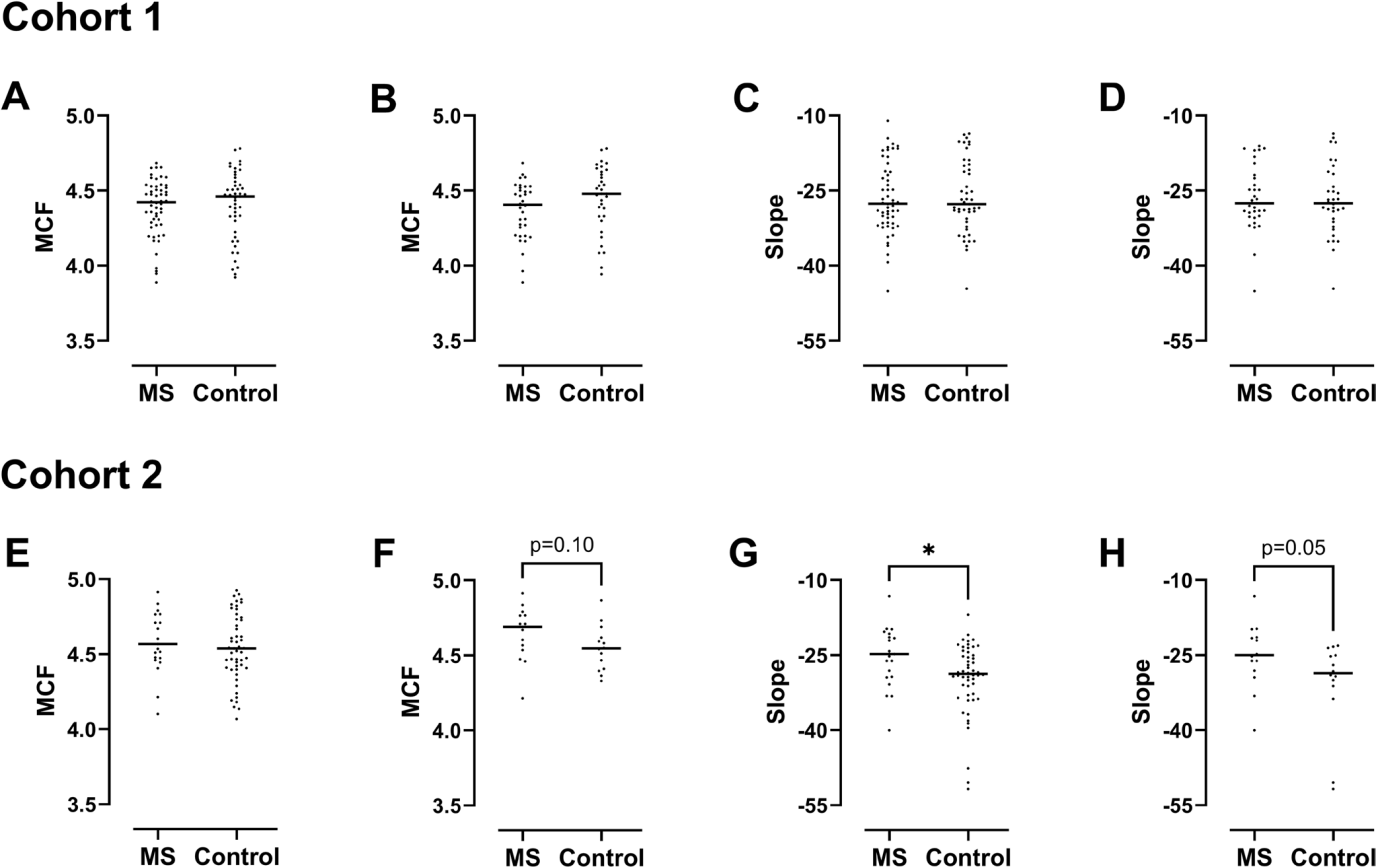
Erythrocyte fragility parameters in Cohort 1 (A–D) and Cohort 2 (E–H): Median corpuscular fragility in all the participants (A, E) and age‐matched subgroup (B, F) and haemolysis curve slope in all the participants (C, G) and age‐matched subgroup (D, H). *p* values are from Mann–Whitney *U* tests. *p < 0.05. MCF, median corpuscular fragility.

Figure 4 presents results for the MCF and the haemolysis curve slope. In Cohort 1, no significant differences in the MCF or haemolysis curve slope were observed between PwPMS and controls, in either the complete‐case or age‐matched analyses. In Cohort 2, there was no difference in the MCF between PwPMS and controls in the complete‐case analysis, but a trend (*p* = 0.10) towards a higher MCF in PwPMS (median = 4.69 g/L) versus controls (median = 4.55 g/L) was observed in the age‐matched analyses. There was a significant group difference (*p* = 0.016) in the haemolysis curve slope between PwPMS (median = −24.8) and controls (median PwPMS = −28.7), which persisted as a trend (*p* = 0.054) in the age‐matched sensitivity analysis (median = −25 in PwPMS and −28.6 in controls). The slope was less steep in PwPMS indicating increased heterogeneity in erythrocyte osmotic fragility compared with controls.

### 3.4 Erythrocyte Fragility Parameters and Whole Brain Volume

MRI data were available at the same timepoint as the osmotic fragility test for 44 PwPMS in Cohort 1. Less than 10% of participants (*n* = 4) showed lesions with post‐contrast enhancement on this first scan. Follow‐up scans were available at 1, 2 and 3 years; 26 participants had imaging data at all four time points.

Linear regression adjusting for age and T2 lesion volume revealed a significant cross‐sectional association between higher MCF and reduced whole brain volume (Table 2). Higher MCF was also associated with reduced whole brain volume 1, 2 and 3 years later. Comparison of the standardized beta coefficients in Table 2 suggests that the MCF had a larger effect size on brain volume than age, and more than half of the effect size of T2 lesion volume. Sensitivity analysis restricted to the subset of participants who had imaging data at all four time points (*n* = 26) showed similar results (Table S1).

**TABLE 2:** Linear regression analysis of the relationship between whole brain volume (dependent variable) and median corpuscular fragility, correcting for potentially confounding variables (age and T2 lesion volume), at study entry and during follow‐up.

	Study entry	1 year	2 years	3 years
*n*	44	30	36	41
Model				
*F* statistic	11.6	6.95	13.6	10.9
Degrees of freedom	3.40	3.26	3.32	3.37
Significance (*p*)	< 0.001	0.001	< 0.001	< 0.001
*R* ^2^ (adjusted)	0.426	0.381	0.520	0.427
Standardized beta coefficients				
MCF	−0.365	−0.449	−0.405	−0.348
Age	−0.207	−0.190	−0.240	−0.255
T2 lesion volume	−0.622	−0.625	−0.673	−0.597
Significance (*p*)				
MCF	0.003	0.006	0.002	0.007
Age	0.085	0.234	0.055	0.041
T2 lesion volume	< 0.001	< 0.001	< 0.001	< 0.001

Abbreviation: MCF, median corpuscular fragility.

The slope of the haemolysis curve was not associated with whole brain volume in the cross‐sectional analysis or during follow‐up (Table 3). A trend was observed, especially at one year, and the regression coefficient was positive, indicating that a reduced whole brain volume was associated with a more negative and therefore steeper slope (i.e., reduced heterogeneity in erythrocyte osmotic fragility). Sensitivity analysis restricted to the subset of participants who had imaging data at all four time points (*n* = 26) showed similar results (Table S2).

**TABLE 3:** Linear regression analysis of the relationship between whole brain volume (dependent variable) and haemolysis curve slope, correcting for potentially confounding variables (age and T2 lesion volume), at study entry and during follow‐up.

	Study entry	1 year	2 years	3 years
*n*	44	30	36	41
Model				
*F* statistic	8.67	5.338	9.063	8.781
Degrees of freedom	3.40	3.26	3.32	3.37
Significance (*p*)	< 0.001	0.005	< 0.001	< 0.001
*R* ^2^ (adjusted)	0.348	0.310	0.409	0.369
Standardized beta coefficients				
Haemolysis curve slope	0.245	0.375	0.238	0.253
Age	−0.195	−0.114	−0.251	−0.259
T2 lesion volume	−0.604	−0.442	−0.620	−0.573
Significance (*p*)				
Haemolysis curve slope	0.056	0.030	0.083	0.054
Age	0.127	0.515	0.073	0.047
T2 lesion volume	< 0.001	0.015	< 0.001	< 0.001

## 4 Discussion

The hypothesis that people with MS have more fragile erythrocytes is based upon prior observations [13, 14, 15] and the previous findings from the MS‐STAT trial cohort which demonstrated an association between elevated serum free Hb and accelerated brain atrophy in SPMS [12]. In the present study imaging data support a potential role for erythrocyte fragility in MS‐related neurodegeneration. In linear regression models, a higher MCF was consistently associated with reduced whole brain volume across multiple timepoints. Two observations are of particular interest. First, the association of MCF with reduced brain volume was substantial, with a magnitude that was larger than that of age and approached that of T2 lesion volume. Second, the baseline MCF was not just associated with reduced brain volume at study entry, but also continued to associate with reduced brain volume up to three years later.

All analyses were adjusted for confounding variables, that is those variables with effects on both dependent and independent variables, namely age and T2 lesion load. The association of age with erythrocyte fragility and brain volume is well documented [43, 45]. T2 lesion volume was included since it impacts brain volume and potentially erythrocyte fragility, the latter since erythrocytes traversing MS plaques are exposed to oxidative stress, inflammatory mediators and haemodynamic shear.

It is important to note that the MCF value integrates fragility across *all* erythrocytes within a patient's sample; therefore, the MCF may lack sensitivity to detect changes in fragility in subpopulations of erythrocytes. Increased osmotic fragility may solely affect ageing cells or those exposed to oxidative stress, and since the haemolysis curve slope is a more sensitive method compared to the MCF [44], it is more likely to detect such subpopulations. In line with this, PwPMS had a less steep haemolysis slope compared with healthy controls (in Cohort 2), demonstrating an increased heterogeneity in erythrocyte osmotic fragility.

In contrast to the results with Cohort 2, erythrocyte fragility parameters did not differ between cases and controls in Cohort 1. The most likely explanation is technical. Building on our experience from Cohort 1, the sampling protocol for Cohort 2 was refined to minimize in vitro haemolysis, which might selectively eliminate the putative erythrocyte subpopulation of interest—namely, the cells susceptible to haemolysis in MS. In Cohort 1, various methods of public and personal transport were utilized to transport the blood sample from the clinic to the laboratory in another building across the city. Since mechanical agitation during transport can lyse erythrocytes [46], this problem was circumvented in Cohort 2 by close juxtaposition of clinical and laboratory facilities within the same building. One of the authors (C.S.) attended each venesection and walked with the sample in a padded container to the laboratory a few minutes away. The time between venesection and osmotic fragility testing was reduced from 3 h to 15 min. Lastly, in Cohort 2 blood tubes were not placed on ice to avoid localized freezing at tube–ice contact points, which can lead to haemolysis [47, 48]. The optimization in Cohort 2 was associated with: (1) a reduction in the level of in vitro haemolysis in isotonic or near‐isotonic buffer concentrations during testing, (2) higher MCF and less steep haemolysis curve slope, compared with Cohort 1, in keeping with preservation of a fragile subpopulation of erythrocytes.

Due to the differences in sample handling mentioned above, it is likely that a subpopulation of fragile erythrocytes (responsible for the less steep slope in Cohort 2) had already undergone haemolysis prior to osmotic challenge in Cohort 1. In keeping with this explanation, the haemolysis curve slope was less steep in PwPMS with reduced brain atrophy in Cohort 1 (Table 3), which is likely to have resulted from loss of fragile erythrocytes before osmotic challenge, paradoxically leading to a more homogenous population. This also explains why the haemolysis curve slope was less sensitive than the MCF in detecting differences between MS patients with varying degrees of atrophy in Cohort 1 (compare Tables 2 and 3), while it was more sensitive in detecting a difference between MS and controls in Cohort 2 (Figure 4). In summary, although the haemolysis curve slope is more sensitive to osmotic fragility changes under optimal sample handling conditions, the MCF is less susceptible to pre‐analytic haemolysis compared with the haemolysis curve slope.

**FIGURE 5:**
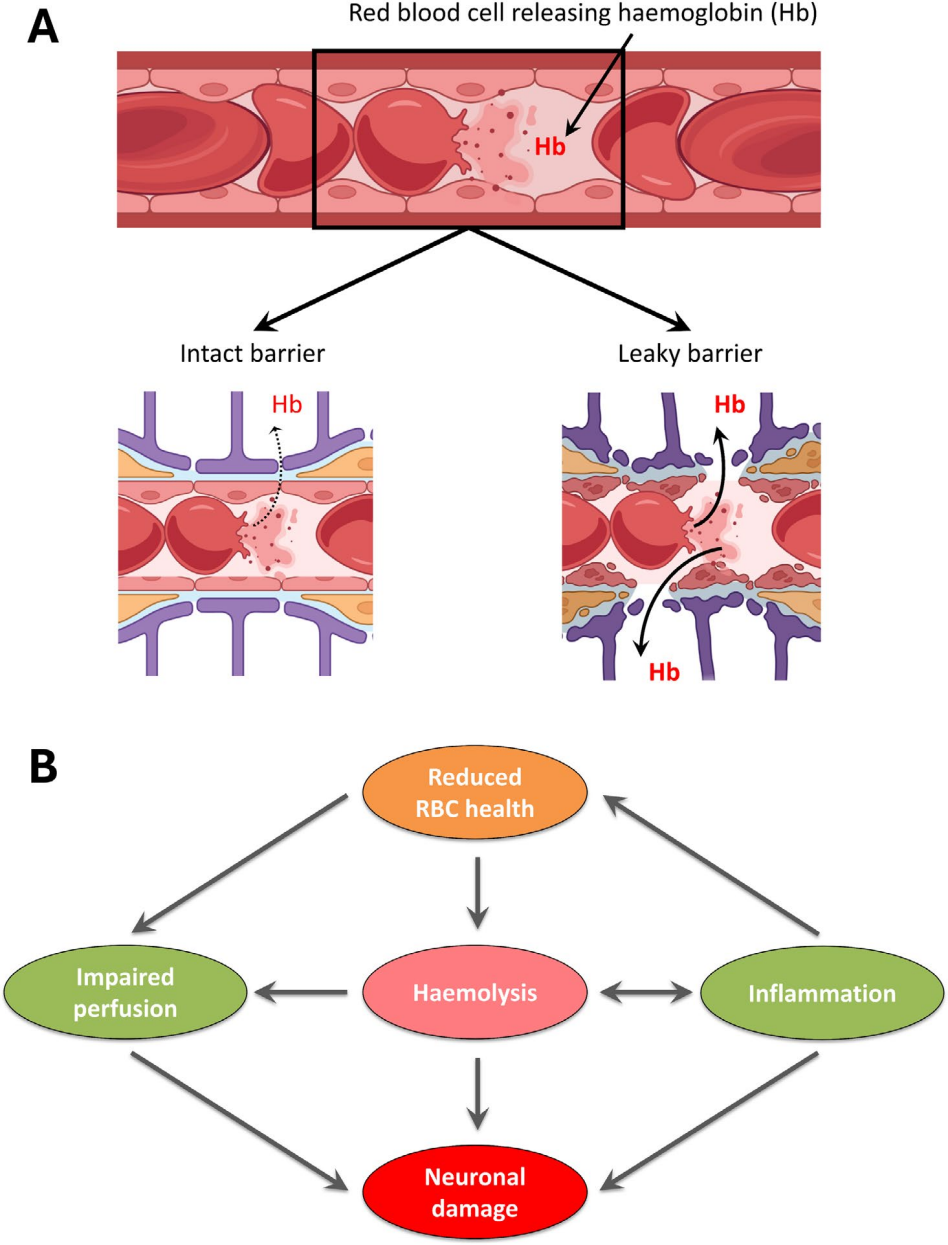
(A) Proposed mechanism by which blood–brain barrier (BBB) dysfunction may potentiate the effects of subclinical haemolysis. Haemolysis may be a feature of a number of common medical conditions, but in the presence of an intact BBB, Hb is unable to enter the brain parenchyma. In people with MS, widespread abnormalities of the BBB, combined with haemolysis, result in permeation of Hb within the CNS, which leads to neurodegeneration and increased iron content. (B) Interplay of inflammation, haemolysis and neurodegeneration.

While the mechanism underlying erythrocyte fragility in MS is unknown, there is evidence that an abnormal inflammatory response in PwMS is present systemically, not just within the CNS [49, 50], and that systemic inflammation causes erythrocyte fragility [43]. Out of the three previously published studies of erythrocyte fragility in MS [13, 14, 15], two suggest that it is more pronounced in ‘active MS’ versus ‘quiescent MS’ [14] and in ‘MS inpatients’ versus ‘MS outpatients’ [15]. We hypothesize that inflammatory activity is more important than the category of MS. Erythrocyte abnormalities are also present in other medical conditions, particularly those associated with inflammation. The key difference, however, is that in PwMS, the effects of this may be particularly detrimental due to the co‐existence of an abnormally high permeability of the blood–brain barrier. Erythrocyte lysis occurring within leaky brain capillaries will result in a localized steep concentration gradient driving Hb into the brain parenchyma affected by MS (Figure 5A). Hence, a substantial relationship between MCF and whole brain volume in PwPMS may occur even if the difference in systemic haemolysis between PwPMS and control individuals is minor and difficult to detect with osmotic fragility testing.

It is important to point out that no causal inferences can be made from this association study. Figure 5B illustrates the potential effects of compromised erythrocyte biology on the brain. Exposure of erythrocytes to inflammation (in the central or systemic circulation) may lead to: (1) impairment of erythrocyte deformability, and hence perfusion, which in turn leads to brain tissue hypoxia; (2) sub‐clinical systemic haemolysis, releasing cell‐free haemoglobin which permeates the leaky blood–brain barrier into the parenchyma to impair neuronal function and/or induce neuronal death, and may exacerbate inflammation. Cell‐free haemoglobin, hypoxia and inflammation all contribute to neurodegeneration. While non‐specific systemic inflammation may result in haemolysis in any individual, the abnormally permeable blood–brain barrier could turn this process into a driver of neuropathology in MS.

Several limitations of this study warrant consideration. The cohorts were relatively small, and osmotic fragility testing was only undertaken at one time point. Osmotic fragility testing does not faithfully replicate the stressors to which erythrocytes are exposed in vivo. Future studies should therefore combine longitudinal assessments of erythrocyte fragility with more physiologically relevant assays, such as ektacytometry, which can recapitulate shear stress and inflammation. Osmotic red blood cell fragility testing is too laborious and sensitive to pre‐analytical conditions to be deployed in clinical situations. Automated ektacytometry is more clinically deployable, and whether it is also as sensitive to pre‐analytical conditions in the MS setting remains to be determined. Focussing on subpopulations of erythrocytes, based for example on markers of cellular senescence or exposure to oxidative stress, may be more informative. Lastly, it would be useful to incorporate additional MR sequences alongside volumetric acquisition to be able to study regional blood–brain permeability and iron content, alongside longitudinal study of free haemoglobin and erythrocyte health.

## 5 Conclusions

We report two main findings. First, we provide evidence that erythrocyte health is impaired in progressive multiple sclerosis and is associated with a reduction in whole brain volume. Second, we demonstrate the critical importance of sample handling to minimize in vitro haemolysis. Further research is needed to disentangle the effects of inflammation, erythrocyte health, haemolysis, blood–brain barrier permeability, iron deposition and neurodegeneration. Advanced neuroimaging techniques combined with functional erythrocyte testing will be helpful to further investigate the role of haemolysis in the pathogenesis of neurodegeneration in progressive MS, which may open avenues for novel therapeutic strategies.

## Author Contributions

**Carmen Jacob:** data curation, investigation, formal analysis, writing – original draft, writing – review and editing. **Thomas E. Williams:** data curation, investigation, formal analysis, writing – original draft, writing – review and editing. **Charlotte M. Stuart:** methodology, data curation, investigation, visualization, validation, project administration, writing – review and editing. **Aviva Witkover:** data curation, investigation, writing – review and editing. **Simon Hametner:** conceptualization, formal analysis, writing – review and editing. **Hans Lassmann:** conceptualization, formal analysis, writing – review and editing. **Charles R. M. Bangham:** conceptualization, writing – review and editing. **Jeremy Chataway:** conceptualization, data curation, investigation, supervision, funding acquisition, writing – review and editing. **Ian Galea:** conceptualization, methodology, formal analysis, funding acquisition, supervision, visualization, project administration, resources, writing – original draft, writing – review and editing.

## Funding

National Institute for Health and Care Research (NIHR), NIHR Health Technology Assessment (HTA) Programme (HTA Project: 15/57/143), UK Multiple Sclerosis Society (68, 83 and 996), National Multiple Sclerosis Society (SI‐1709‐29165 and RG‐1607‐24869) and the Rosetrees trust (PGL‐pre2019/10013).

## Conflicts of Interest

Jeremy Chataway has received support from the Health Technology Assessment (HTA) Programme (National Institute for Health and Care Research; NIHR), the UK MS Society, the US National MS Society, and the Rosetrees Trust. Supported in part by the NIHR University College London Hospitals Biomedical Research Centre, London, UK. He has been local principal investigator for a trial in multiple sclerosis funded by MS Canada; local principal investigator for commercial trials funded by Ionis and Roche; and has taken part in advisory boards or consultancy for Biogen, Contineum Therapeutics, FSD Pharma, InnoCare, Pheno Therapeutics, and Roche.

Ian Galea has received research support from the National Institute for Health and Care Research, the UK MS Society, Medical Research Council, the Rosetrees Trust, the Geoff Smith Foundation in aid of Multiple Sclerosis, the University of Southampton, Wessex Medical Research, Independent Research Fund Denmark, Kedrion, Alzheimer's Society, The Binding Site, Bio Products Laboratory Limited, Gilead Sciences, Bio Products Laboratory Limited, Merck‐Serono, The Gerald Kerkut Charitable Trust, Association of British Neurologists, Evgen Pharma, Engineering and Physical Sciences Research Council, Alzheimer's Research UK, University of Southampton, Smile4Wessex, Academy of Medical Sciences/Wellcome Trust, and Peel Medical Research Trust. He has received travel support from Teva and Novartis, consultancy fees from CSL Behring and speaker honoraria from Gilead Sciences and Novartis.

## Supporting information


**Table S1:** | Linear regression analysis of the relationship between whole brain volume (dependent variable) and median corpuscular fragility, correcting for potentially confounding variables (age and T2 lesion volume), for participants (*n* = 26) with MRI data at all time points, i.e., at study entry and over the following three years.
**Table S2:** | Linear regression analysis of the relationship between whole brain volume (dependent variable) and haemolysis curve slope, correcting for potentially confounding variables (age and T2 lesion volume), for participants (*n* = 26) with MRI data at all time points i.e., study entry and over the following three years.

## Data Availability

The data that support the findings of this study are available from the corresponding author upon reasonable request, subject to institutional and ethical approvals.
