# Supplementary material for: Erythrocyte Fragility in Progressive Multiple Sclerosis
Source: Eur J Neurol. 2026 Apr 17;33(4):e70595. doi: 10.1111/ene.70595 (PMC13088977; doi:10.1111/ene.70595)
Supplement: Supplementary file 1 — Table S1: | Linear regression analysis of the relationship between whole brain volume (dependent variable) and median corpuscular fragility, correcting for potentially confounding variables (age and T2 lesion volume), for participants (n = 26) with MRI data at all time points, i.e., at study entry and over the following three years. Table S2: | Linear regression analysis of the relationship between whole brain volume (dependent variable) and haemolysis curve slope, correcting for potentially confounding variables (age and T2 lesion volume), for participants (n = 26) with MRI data at all time points i.e., study entry and over the following three years. [file ENE-33-e70595-s001.docx]

Supplementary material for:

Erythrocyte fragility in progressive multiple sclerosis

Carmen Jacob^1,2,^*, Thomas E. Williams^3,^*, Charlotte M. Stuart^1^, Aviva Witkover^4^, Simon Hametner^6^, Hans Lassmann^7^, Charles R. M. Bangham^4^, Jeremy Chataway^3,5,#^, Ian Galea^1,2,#^

1. Clinical Neurosciences, Clinical & Experimental Sciences, Faculty of Medicine, University of Southampton, Southampton, UK
2. Wessex Neurological Centre, Southampton General Hospital, University Hospital Southampton NHS Foundation Trust, Southampton, UK
3. Queen Square Multiple Sclerosis Centre, Department of Neuroinflammation, UCL Queen Square Institute of Neurology, Faculty of Brain Sciences, University College London, London, UK
4. Department of Infectious Diseases, Faculty of Medicine, Imperial College London, London, United Kingdom
5. National Institute for Health Research, Biomedical Research Centre, University College London Hospitals, London, United Kingdom
6. Division of Neurochemistry and Neuropathology, Medical University of Vienna, Vienna, Austria
7. Department of Neuroimmunology, Center for Brain Research, Medical University of Vienna, Vienna, Austria

**Supplementary Table S1**

**Supplementary Table S2**

**TABLE S1** | Linear regression analysis of the relationship between whole brain volume (dependent variable) and median corpuscular fragility, correcting for potentially confounding variables (age and T2 lesion volume), for participants (n=26) with MRI data at all time points, i.e. at study entry and over the following three years.

|  | **Study entry** | **1 year** | **2 years** | **3 years** |
| --- | --- | --- | --- | --- |
| **Model** |  |  |  |  |
| F statistic | 5.10 | 6.77 | 6.52 | 6.53 |
| Degrees of freedom | 3,22 | 3,22 | 3,22 | 3,22 |
| Significance (p) | 0.008 | 0.002 | 0.003 | 0.003 |
| R² (adjusted) | 0.330 | 0.409 | 0.398 | 0.399 |
| **Standardised beta coefficients** |  |  |  |  |
| MCF | -0.346 | -0.383 | -0.360 | -0.350 |
| Age | -0.273 | -0.332 | -0.296 | -0.242 |
| T2 lesion volume | -0.593 | -0.632 | -0.634 | -0.627 |
| **Significance (p)** |  |  |  |  |
| MCF | .059 | 0.028 | 0.040 | 0.044 |
| Age | .134 | 0.058 | 0.087 | 0.154 |
| T2 lesion volume | .002 | <0.001 | <0.001 | <0.001 |

Abbreviation: MCF, median corpuscular fragility

**TABLE S2** | Linear regression analysis of the relationship between whole brain volume (dependent variable) and haemolysis curve slope, correcting for potentially confounding variables (age and T2 lesion volume), for participants (n=26) with MRI data at all time points i.e. study entry and over the following three years.

|  | **Study entry** | **1 year** | **2 years** | **3 years** |
| --- | --- | --- | --- | --- |
| **Model** |  |  |  |  |
| F statistic | 5.16 | 6.14 | 5.75 | 5.35 |
| Degrees of freedom | 3,22 | 3,22 | 3,22 | 3,22 |
| Significance (p) | 0.008 | 0.003 | 0.005 | 0.006 |
| R² (adjusted) | 0.333 | 0.381 | 0.363 | 0.343 |
| **Standardised beta coefficients** |  |  |  |  |
| Haemolysis curve slope | 0.366 | 0.358 | 0.318 | 0.275 |
| Age | -0.211 | -0.279 | -0.261 | -0.228 |
| T2 lesion volume | -0.418 | -0.454 | -0.469 | -0.481 |
| **Significance (p)** |  |  |  |  |
| Haemolysis curve slope | 0.055 | 0.051 | 0.085 | 0.143 |
| Age | 0.264 | 0.133 | 0.157 | 0.215 |
| T2 lesion volume | 0.029 | 0.015 | 0.013 | 0.012 |
